# Polysialic acid restrains inflammatory monocyte maturation

**DOI:** 10.3389/fimmu.2025.1656087

**Published:** 2025-10-20

**Authors:** Ingredy Passos, Benjamin Peschke, Shrey Gandhi, Judith Derdelinckx, Louisa Müller-Miny, Harald Neumann, Jan D. Lünemann, Christian W. Keller

**Affiliations:** 1 Department of Neurology with Institute of Translational Neurology, University Hospital Münster, Münster, Germany; 2 Laboratory of Neuroinflammation, Institute of Experimental Immunology, University of Zurich, Zurich, Switzerland; 3 Department of Immunology, University Hospital Münster, Münster, Germany; 4 Division of Genetic Epidemiology, Institute of Human Genetics, University of Münster, Münster, Germany; 5 Department of Neurology, Faculty of Medicine and Health Sciences, Antwerp University Hospital, Antwerp, Belgium; 6 Laboratory of Experimental Hematology, Vaccine and Infectious Disease Institute (VaxInfectio), Faculty of Medicine and Health Sciences, University of Antwerp, Antwerp, Belgium; 7 Institute of Reconstructive Neurobiology, Medical Faculty and University Hospital of Bonn, University of Bonn, Bonn, Germany; 8 Department of Neurology and Neurophysiology, University Medical Center Freiburg, Freiburg, Germany

**Keywords:** polysialic acid, Siglec receptors, myeloid cells, neuroinflammation, autoimmunity, multiple sclerosis, monocyte maturation

## Abstract

Sialic acids are widely distributed monosaccharides in the central nervous system (CNS), where they are predominantly found as terminal sialic acid residues, as well as in di-, oligo-, and polysialic forms on the glycocalyx, collectively contributing to the development, resilience, and long-term integrity of the CNS. Harnessing sialic acid–binding immunoglobulin-like lectin (Siglec) receptors by α2.8-linked polysialic acids has been shown to modulate immune responses. In this study, murine and human monocytes were exposed to α2.8-linked low molecular weight polysialic acid (α2.8-polySIA) in vitro, followed by phenotypic, functional, and transcriptomic analyses using flow cytometry and RNA sequencing; therapeutic efficacy was assessed in mice with experimental autoimmune encephalomyelitis (EAE), a pre-clinical model of multiple sclerosis (MS). Here, we report that α2.8-polySIA inhibits toll-like receptor-induced phenotypical and functional maturation of murine and human monocytes into pro-inflammatory effector cells equipped with operational antigen-presenting machinery. Moreover, RNA sequencing analyses revealed a shift towards a regulatory phenotype in human myeloid cells exposed to α2.8-polySIA. Finally, therapeutic treatment with α2.8-polySIA led to a milder disease course in EAE mice. Thus, by tuning myeloid cell phenotype *in vivo*, the therapeutic application of polysialic acid may offer a novel approach to modulate myeloid-driven inflammation in CNS autoimmunity.

## Introduction

Multiple sclerosis (MS) is a chronic inflammatory and neurodegenerative disease of the central nervous system (CNS). Currently, available disease-modifying therapies, all of which have immunomodulatory and/or immunosuppressive properties, mainly improve the course of relapsing-remitting MS (RRMS), the most common disease phenotype. However, their effect on clinical disability progression is modest (1, 2). In addition, currently approved therapies fail to show efficacy in the later secondary progressive phase, underscoring the need for safe treatments that address both inflammatory and neurodegenerative mechanisms (1, 2).

Sialic acid is the collective name of a family of 9-carbon carboxylated sugars which are usually found as terminal residues at the non-reducing end of sugar chains on glycoproteins and glycolipids. The CNS shows the highest expression of sialic acid (3). A distinctive property of sialic acid is its ability to form homo-oligo/polymeric structures such as di-, oligo-, and polysialic acids. Polysialic acid is broadly expressed in the developing and injured vertebrate CNS, where it plays essential roles in repair processes including cell migration, axon guidance, and synaptic plasticity (3). Accumulating data indicate that soluble polysialic acid additionally modulates immune system components and has powerful anti-inflammatory properties (4–8).

In MS, polysialylated neural cell adhesion molecule (PSA-NCAM), normally absent from adult axons, becomes re-expressed on demyelinated axons within plaques, but not on remyelinated axons in shadow plaques (9). Likewise, analyses of the subventricular zone (SVZ) in MS show a 2–3 fold increase in cell density and proliferation, with PSA-NCAM^+^ early glial progenitors being up to 8-fold more numerous in active and chronic-active lesions compared with chronic silent lesions, shadow plaques, or normal-appearing white matter (10). In experimental autoimmune encephalomyelitis (EAE), an animal model for MS, PSA-NCAM^+^ neural progenitors are mobilized from the SVZ into inflamed white matter, where they contribute to glial lineages (11). In addition, microglia themselves contain an intrinsic pool of polysialylated proteins that is translocated and released upon inflammatory activation, with released polySia attenuating microglial responses via Siglec-E, suggesting dynamic regulation of polySia during CNS inflammation (12).

Immune functions of sialic acids can be generally summarized into two categories: I) The highly anionic sialic acids act as a biological safeguard that shields recognition sites from the immune system. II) Sialic acids together with downstream sugar moieties can also function as biological “self” recognition sites through ligation of sialic acid binding immunoglobulin-like lectin (Siglec) receptors, predominantly expressed on innate immune cells (13). Immune suppressive signaling of Siglecs is mediated mostly via ITIMs (immunoreceptor tyrosine-based inhibition motif), while only a few Siglecs signal via ITAMs (immunoreceptor tyrosine-based activation motifs). In line with these findings, nanoparticles decorated with α2.8-linked di-sialic acid were shown to block the production of LPS-induced inflammatory cytokines in a Siglec-dependent manner and to be therapeutically beneficial in LPS-induced murine models of sepsis and acute respiratory distress syndrome (4). SIGLEC-7, SIGLEC-9, and SIGLEC-11 are polySIA-binding Siglecs expressed in humans (13, 14). In humanized transgenic mice expressing SIGLEC-11 on mononuclear phagocytes, α2.8-linked low molecular weight polySIA inhibited complement activation and tumor necrosis factor-α (TNF-α) production and protected from inflammatory retinal damage in an animal model of age-related macular degeneration (AMD) (5, 6). Low molecular weight polysialic acid also prevented the lipopolysaccharide-induced inflammatory dopaminergic neurodegeneration in humanized SIGLEC-11 transgenic mice (15). Additionally, the loading of sialic acid-decorated antigens has shown to inhibit murine T_H_1 and T_H_17 effector cell responses and to promote the generation of antigen-specific regulatory T cells (Tregs) via dendritic cells (DCs) (16).

Monocytes are circulating myeloid immune precursor cells originating from the bone marrow. Mature monocytes patrol the circulation and may be recruited to peripheral sites of inflammation to differentiate on-site into monocyte-derived effector cells (17, 18). In case of the CNS, classical monocytes, initiated by a yet unknown trigger, differentiate into antigen-presenting cells (APCs) (19). Monocyte differentiation under inflammatory conditions *in vivo* is likely to be site-specific and mediated by various signals (19–22).

While EAE is highly dependent on and initiated by dysregulated, autoantigen reactive CD4^+^ T helper cells, CNS tissue damage, i.e. demyelination, is predominantly executed by CD11c^neg^Ly6C^+^ monocytes, precursors of CNS-invading monocyte-derived DCs rather than through CNS-resident antigen-presenting cells such as microglia or CNS autochthonous DCs (23–27). Monocytes are characterized by high degrees of plasticity and, upon appropriate pattern recognition, can give rise to cells depicting hallmarks of either macrophages or DCs (22, 28). Therefore, the transition of immature monocytes to mature monocyte-derived APCs, e.g. via ligation of Toll-like receptor (TLR)ligand 4 via lipopolysaccharide (LPS), involves copious changes in phenotype and functionality (29, 30). These matured APCs are characterized by the expression of high levels of MHC class II, costimulatory molecules (such as CD40, CD80, CD86), chemokines, and cytokines, in stark contrast to their immature counterparts which depict low expression of these molecules and high levels of phagocytic activity and associated receptors (29–31).

Here we report that exposure to α2.8-polySIA robustly abrogates monocyte maturation into monocyte-derived APCs and treatment with α2.8-polySIA early in the disease course of EAE ameliorates further disease progression.

## Materials and methods

### Mice

Wild-type C57BL/6 mice were purchased from Janvier Labs. All animals were bred and housed in the University of Zurich and University of Münster animal facilities in individually ventilated cages on a 12 h light/dark cycle with food and water available *ad libitum* according to institutional guidelines, as well as Swiss and German animal laws. All animal protocols were approved by and conducted in accordance with the cantonal veterinary office of the canton of Zurich, Switzerland (ZH190/17) and the State Agency for Nature, Environment and Consumer Protection North Rhine-Westphalia, Germany (81**-**02.04.2019.A413).

### Induction of experimental autoimmune encephalomyelitis

EAE was induced as previously described (32). In brief, wild-type C57BL/6 mice were immunized with 200 μg of MOG_35_**_–_**_55_ (MEVGWYRSPFSRVVHLYRNGK; Genscript, RP10245) emulsified in CFA (BD Difco, 263810) and treated with 200 ng of pertussis toxin (List Biological Laboratories, 179B) i.p. on the day of immunization followed by a second treatment with 200 ng of pertussis toxin on day 2. Clinical manifestations of EAE and weight loss were monitored and documented daily. Mice were scored as follows: 0 = no detectable signs of EAE, 0.5 = distal limp tail, 1 = complete limp tail, 1.5 = limp tail and hindlimb weakness, 2 = unilateral partial hindlimb paralysis, 2.5 = bilateral partial hindlimb paralysis, 3 = complete bilateral hindlimb paralysis, 3.5 = complete bilateral hindlimb paralysis and partial forelimb paralysis, 4 = moribund (animal unable to move due to tetra paralysis), 5 = animal found dead. The last documented score of euthanized or dead animals was carried forward for statistical analysis.

### Treatment with α2.8-linked polySIA

Low molecular polysialic acid (α2.8-linked polySIA) was obtained as previously described (15). The α2.8-linked polySIA was applied repeatedly i.p. (10 µg/g body weight over 4 consecutive days) after an EAE score of 2 (unilateral partial hind limp paralysis) was reached. PBS was used as vehicle control.

### Flow cytometry

Single-cell suspensions were pre-incubated with Fc receptor block (20 min, 4 °C, 22.4 μg/ml; clone: 2.4 G2; Bio X Cell, CUS-HB-197) followed by staining with the LIVE/DEAD Fixable Aqua Dead Stain Kit (Thermo Fisher Scientific L34957) in PBS at 4 °C in the dark. Cells were stained with fluorochrome-labeled antibodies in FACS buffer (0.5% BSA [Sigma, 05470] and 0.01% NaN3 [Sigma, S8032–25 G] in PBS) for 30 min at 4 °C in the dark. Samples were acquired and recorded using CytExpert 2.3 software on a CytoFLEX flow cytometer (Beckman Coulter, Brea, CA). Analysis was performed using the FlowJo software v9.3.1 and v10 (Tree Star).

### Magnetic activated cell sorting

All magnetic activated cell sorting (MACS) procedures were carried out using magnetic MicroBeads from Miltenyi and the autoMACS Pro Separator (130-092-545; Miltenyi) in accordance with the provider**’**s protocol recommendations.

### Generation and LPS stimulation of BMDCs

For the culture of bone-marrow-derived dendritic cells (BMDCs), monocytes were isolated from bone marrow cells of C57BL/6 animals using Anti-biotin MACS-Beads (Miltenyi Biotec, 130-090-485) and biotinylated anti-Ly6c antibody (clone HK1.4; Biolegend, 128004), following the manufacturer’s recommendations. Monocytes (6×10^4^/well) were plated in 96-well U-bottom plates and maintained in RPMI 1640 medium (10% FCS + 1% Pen/Strep + 50 µM β-ME) supplemented with 22 ng/ml granulocyte-macrophage colony-stimulating factor (GM-CSF) and 5.5 ng/ml interleukin (IL)-4 in the presence or absence of α2.8-linked polySIA at a concentration of 1.5 µM for a period of 3 days at 37 °C and 5% CO_2_. PBS was used as the vehicle and negative control in all experiments. After 3 days, half of the supernatant was carefully removed and stored for cytokine analysis, and fresh medium was added to the wells. On day 5, immature moDCs were stimulated with LPS 500 ng/ml as a positive control for activation, in the presence or absence of polySIA 1.5 µM. PBS-treated cells without LPS served as unstimulated negative controls. After 24 hours of stimulation, supernatants were collected for cytokine analysis, and cells were harvested for flow cytometry staining (**Supplementary Figure S1**). After 24 hours of stimulation, supernatants were collected for cytokine analysis, and cells were harvested for flow cytometry staining. The concentration of polySIA (1.5 μM) used in these experiments was chosen based on prior studies (5, 6). The panel used for flow cytometry analysis included the following markers: MHCII PB450 (Dilution 1:200; Clone M5/114.15. 2; cat 107620; BD Biosciences, Germany); CD11c PE/Cy7 (Dilution 1:400; Clone N418; cat 117318; BioLegend, Germany); CD80 FITC (Dilution 1: 25; Clone 16-10A1; cat 104706; BioLegend, Germany); CD86 AF700 (Dilution 1:25; Clone PO3; cat 105122; BioLegend, Germany); CD40 APC (Dilution 1:50; Clone 3/23; cat 124612 BioLegend, Germany); and Zombie Aqua - Live/Dead KO252 (Dilution 1:200; cat 423102; BioLegend, Germany).

### Generation and LPS stimulation of moDCs

For the culture of human monocyte-derived dendritic cells (moDCs), monocytes were isolated from PBMCs from healthy donors using the *Pan Monocytes Isolation Kit* from Miltenyi Biotec, following the manufacturer’s recommendations. Monocytes (5×10^5^/well) were plated on 48-well plates and maintained in RPMI 1640 medium (10% FCS + 1% Pen/Strep + 50µM β-ME) supplemented with 22 ng/ml human GM-CSF and 5.5 ng/ml human IL-4 in the presence or absence of α2.8-linked polySIA at a concentration of 1.5 µM for a period of 3 days at 37 °C and 5% CO_2_. PBS was used as the vehicle and negative control in all experiments. After 3 days, half of the supernatant was carefully removed and stored for cytokine assay, and fresh medium was added to the wells. On day 5, immature moDCs were stimulated with LPS 500 ng/ml as a positive control for activation, in the presence or absence of polySIA 1.5 µM. PBS-treated cells without LPS served as unstimulated negative controls. After 24 hours of stimulation, supernatants were collected for cytokine analysis, and cells were harvested for flow cytometry staining (**Supplementary Figure S2**). Panel used for flow cytometry analysis included the following markers: CD14 PB450 (Dilution 1:50; Clone M5E2; cat 558121; BD Biosciences, Germany); CD209 FITC (Dilution 1:50; Clone 9E9A8; cat 330104; BioLegend, Germany); HLA-DR PE (Dilution 1:50; Clone L243; cat 307605; BioLegend, Germany); CD80 V660 (Dilution 1: 50; Clone 2D10; cat 305227; BioLegend, Germany); CD83 PC5 PerCP (Dilution 1:50; Clone HB15e; cat 305320; BioLegend, Germany); CD86 APC (Dilution 1:50; Clone BioLegend, Germany); Zombie Aqua - Live/Dead KO252 (Dilution 1:200; cat 423102; BioLegend, Germany) and CD11c PC7 (Dilution 1:25; BioLegend, Germany).

### RNA-sequencing

#### Library preparation

Total RNA was isolated using the Direct-zol RNA Microprep Kit (Zymo Research Europe GmbH, Freiburg, Germany) including a DNase digestion step. The integrity of the total RNA was assessed by means of Bioanalyzer RNA 6000 Nano Kit (Agilent, Santa Clara, California) and then the RNA was used for rRNA depletion using the NEBNext^®^ rRNA Depletion Kit (NEB) and subsequent directional library preparation (NEBNext Ultra II RNA Library Prep Kit, New England Biolabs, Inc., Ipswich, Massachusetts). The quality of the resulting NGS library was determined by means of the Bioanalyzer High Sensitivity DNA Kit (Agilent, Santa Clara, California). Equimolar library pools based on the library quantification results of the NEBNext Library Quant Kit for Illumina (New England Biolabs, Inc., Ipswich, Massachusetts) were sequenced in a paired-end mode 111 cycles on a NextSeq 2000 system (Illumina, Inc., San Diego, California) using v3 chemistry.

#### Read mapping and transcriptome quantification

The sequencing reads from each RNA-Seq library were subjected to stringent quality control. Using *Trimmomatic* (v0.36), adapters and all the reads with average Phred quality score < 20 were removed (33)⁠. Poor quality bases were trimmed using the parameter SLIDINGWINDOW:4:15 and only reads longer than 35 bases were retained for further analysis. Processed reads from each sample were then aligned to the human reference genome (Ensembl version 90) using the STAR aligner (v2.5.3a) (34). The mapped reads from each sample were independently assembled into transcripts and quantified using StringTie (v1.3.4d) guided by the reference genome annotations (35)⁠.

#### Expression quantification and differential expression analysis

The read quantifications were then imported into R and *DESeq2* (v1.22.2) (36)⁠ and were used to identify differentially expressed genes (DEGs). Genes with total counts across samples less than 5 and mean read count <2 were excluded from the analysis. Only genes with adjusted p-value < 0.05 (Benjamini-Hochberg) were considered as significantly differentially expressed (**Supplementary Tables 1**, **2**).

#### Enrichment network analysis

To explore the processes that are differentially enriched across the sample groups, enrichment analysis was performed separately for significantly upregulated and downregulated genes using *g:Profiler* (37) with default parameters.

#### Mixed lymphocyte reaction assay

For the mixed lymphocyte reaction assay (MLRA), CD3^+^ T cells were isolated from PBMCs of a healthy donor using human CD3 MicroBeads from Miltenyi Biotec (cat 130-050-101), according to the manufacturer’s recommendations. CD3^+^ T lymphocytes were labelled with CFSE Cell Division Tracker kit (cat 423801; Biolegend, Germany) and plated together with moDCs (pretreated or untreated with polySIA as described above) in a 10:1 ratio, 5×10^4^ T cells and 5×10^3^ moDCs, in 96 well deep U plates. T cells plated without moDCs were used as control. For positive control, cells were treated with PMA 50 ng/ml and ionomycin 1µg/ml. After 5 days of incubation, the supernatant was stored for cytokine analysis and cells were labeled and analyzed by flow cytometry (**Supplementary Figure S3**). Panel used for flow cytometry analysis: Zombie Aqua - Live/Dead KO252 (Dilution 1:200; cat 423102; BioLegend, Germany); CD4 PE (Dilution 1:200); CD8 APC (Dilution 1:200); CD3 AF700 (Dilution 1:100). For proliferation analysis (CFSE), FITC channel was used as recommended by the manufacturer.

#### Statistical analysis

Statistical tests applied are indicated in the respective figure legends. Unpaired, two-tailed student t-test and two-way ANOVA were performed. A P-value < 0.05 was considered statistically significant. The asterisks depicted in the figures translate into the following grouping: * p < 0.05, ** p < 0.01, *** p < 0.001, **** p < 0.0001 in comparison with the respective control group (DC without polySIA or LPS treatment). The hashes depicted in the figures translate into the following grouping: # p < 0.05, ## p < 0.01, ### p < 0.001, #### p < 0.0001 in comparison with the LPS stimulated group (without polySIA treatment). All quantitative analyses were performed with GraphPad Prism v9.0.2 for Mac OSX (GraphPad Software, Inc). All statistics related to bulk Sequencing were performed in R Studio as described above.

## Results

**Figure 1:**
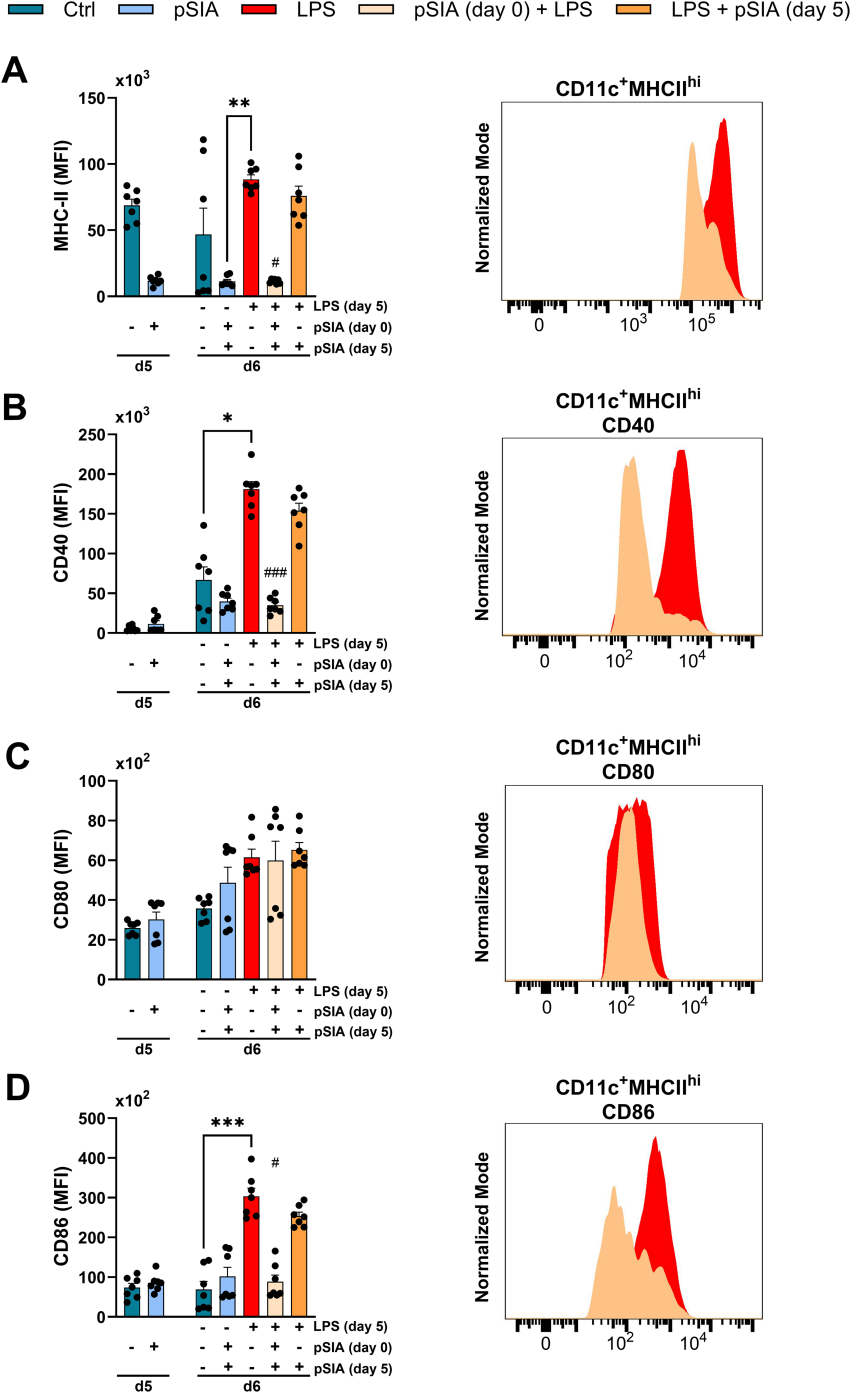
Dynamics of immune phenotype of α2.8-polySIA-treated murine bone marrow-derived DCs. Data are expressed as the mean fluorescent intensity (MFI) ± SEM for **(A)** MHCII, **(B)**, CD40 **(C)**, CD80, and **(D)** CD86. All gated on viable CD11c^+^MHCII^+^ cells. Cells treated with PBS alone represent the negative control, while cells stimulated with LPS alone serve as the positive control for activation. Right panels display representative histograms of each marker in pSIA (day 0) + LPS and LPS (without pSIA) conditions, using normalized mode to compare expression profiles. Statistical comparisons were performed using the Kruskal–Wallis test with Dunn’s *post-hoc* analysis. Significance: *, p < 0.05, **, p < 0.01, ***, p < 0.001 vs. Control group. #, p < 0.05, ###, p < 0.001 vs. LPS group. Pooled data from two experiments is depicted. LPS, lipopolysaccharide; pSIA, α2.8-polySIA.

To investigate how exposure to α2.8-linked polySIA (α2.8-polySIA) impacts the maturation and activation of monocytes *in vitro*, murine Ly6C^+^ bone marrow-derived cells were incubated with GM-CSF and IL-4 in the presence or absence of α2.8-polySIA. On day 5, bone marrow-derived cells were incubated with LPS for activation. We detected a strong reduction in LPS-mediated upregulation of MHC class II, CD40, and CD86 (but not CD80) surface expression upon early exposure to α2.8-polySIA (**Figures 1A-D**). These effects were highly dependent on early (day 0) exposure to α2.8-polySIA, while incubation at a later time point (day 5) only led to minor reductions in MHC class II^+^ CD40 and CD86 surface expression. Treatment with polySIA in the presence of LPS also significantly reduced LPS-induced secretion of pro-inflammatory cytokines and simultaneously increased production of the anti-inflammatory cytokine IL-10, consistent with a more tolerogenic shift in monocyte-derived cells (**Supplementary Figure S4**).

**Figure 2:**
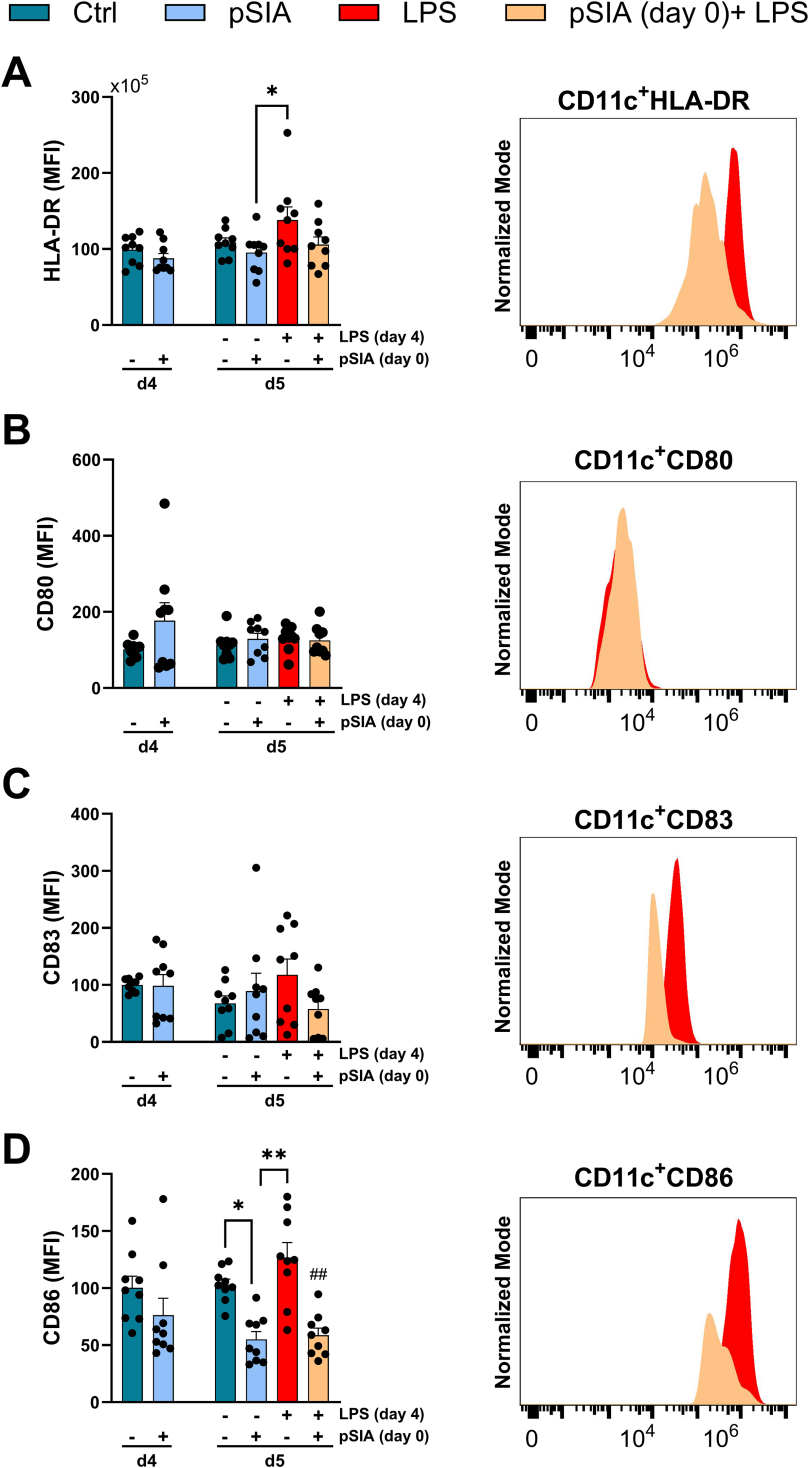
Dynamics of immune phenotype of α2.8-polySIA-treated human monocyte-derived DCs. Data are expressed as the mean fluorescent intensity (MFI) ± SEM for **(A)** HLA-DR, **(B)** CD80, **(C)** CD83, and **(D)** CD86. All gated on viable CD11c^+^CD209^+^ cells. Cells treated with PBS alone represent the negative control, while cells stimulated with LPS alone serve as the positive control for activation. Right panels display representative histograms of each marker in pSIA (day 0) + LPS and LPS (without pSIA) conditions, using normalized mode to compare expression profiles. Statistical comparisons were performed using the Kruskal–Wallis test with Dunn’s *post-hoc* analysis. Significance: *, p < 0.05, **, p < 0.01 vs. Control group. ##, p < 0.01 vs. LPS group. Pooled data from three experiments is depicted. LPS, lipopolysaccharide; pSIA, α2.8-polySIA.

We next analyzed the effects of α2.8-polySIA on the maturation and activation of human monocytes. To this end, PBMCs derived from healthy blood donors were simultaneously enriched for classical (CD14^++^CD16^–^), non-classical (CD14^+^CD16^++^), and intermediate (CD14^++^CD16^+^) monocytes and surface expression profiles of HLA-DR, CD80, CD83, and CD86 were evaluated following standard maturation and activation protocols (**Figures 2A-D**). We observed a trend towards lower HLA-DR^+^ and CD83 surface expression upon early (day 0) α2.8-polySIA exposure and significant impairment of LPS-mediated upregulation of CD86. In contrast, we did not observe any LPS-dependent upregulation in CD80 surface expression (**Figure 2B**). Again, treatment with polySIA in the presence of LPS lead to a shift in the secreted cytokine pattern (**Supplementary Figure S5**).

Thus, early exposure of murine and human monocytes to α2.8-polySIA leads to impaired maturation and activation through diminished surface expression of MHC class II and co-stimulatory molecules.

**Figure 3:**
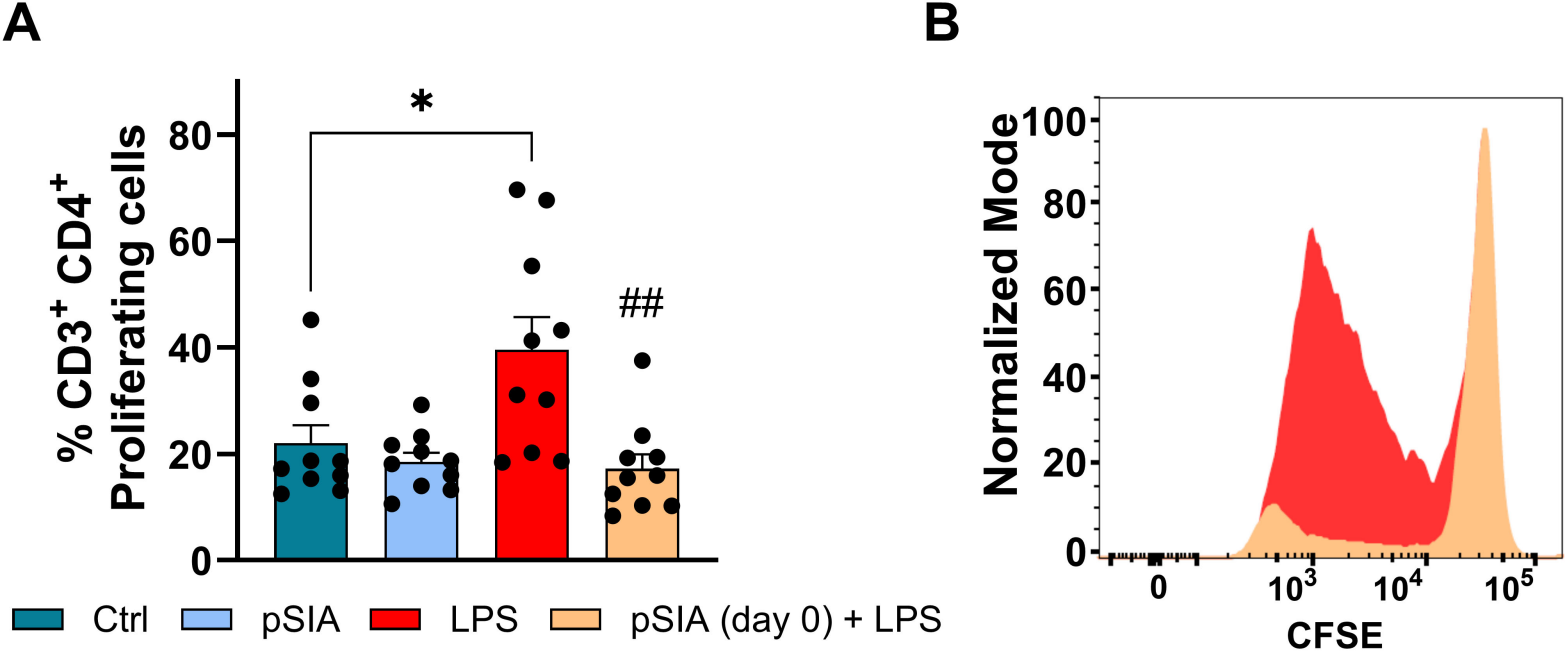
Allogeneic proliferation of CD3^+^ CD4^+^ T cells co-cultured with human monocyte-derived DCs under different treatment conditions at a 10:1 T cell to DC ratio. **(A)** Percentage of proliferating CD3^+^ CD4^+^ T cells, reflecting the stimulatory capacity of DCs. Four experimental groups were included: untreated DCs (Control; PBS-treated, negative control), DCs treated with α2.8-polySIA alone from day 0 (pSIA), DCs stimulated with LPS alone (LPS; positive control for activation), and DCs treated with both α2.8-polySIA from day 0 and LPS (pSIA + LPS). Results are presented as mean ± SEM. Statistical analysis was performed using One-Way ANOVA with Holm–Sidak *post-hoc* test. *, p < 0.05 vs. Control group; ##, p < 0.01 vs. LPS group. **(B)** Representative histogram of CFSE dilution in CD3+ CD4+ T cells co-cultured with DCs from the LPS and pSIA + LPS groups, shown in normalized mode for direct comparison of proliferation profiles. Data are pooled from three independent experiments. LPS, lipopolysaccharide; pSIA, α2.8-polySIA.

We now hypothesized that the functional capacity of monocyte-derived antigen-presenting cells to facilitate CD4^+^ T cell proliferation is compromised upon exposure to α2.8-polySIA. To test our hypothesis, we performed a mixed lymphocyte reaction assay (MLRA) during which CD4^+^ T cells from one donor will proliferate in the presence of antigen-presenting cells from an allogeneic donor by virtue of HLA mismatch recognition. In line with our hypothesis, we observed impaired CD4^+^ T cell proliferation upon co-coculture with α2.8-polySIA pre-exposed antigen-presenting cells (**Figures 3A, B**).

**Figure 4:**
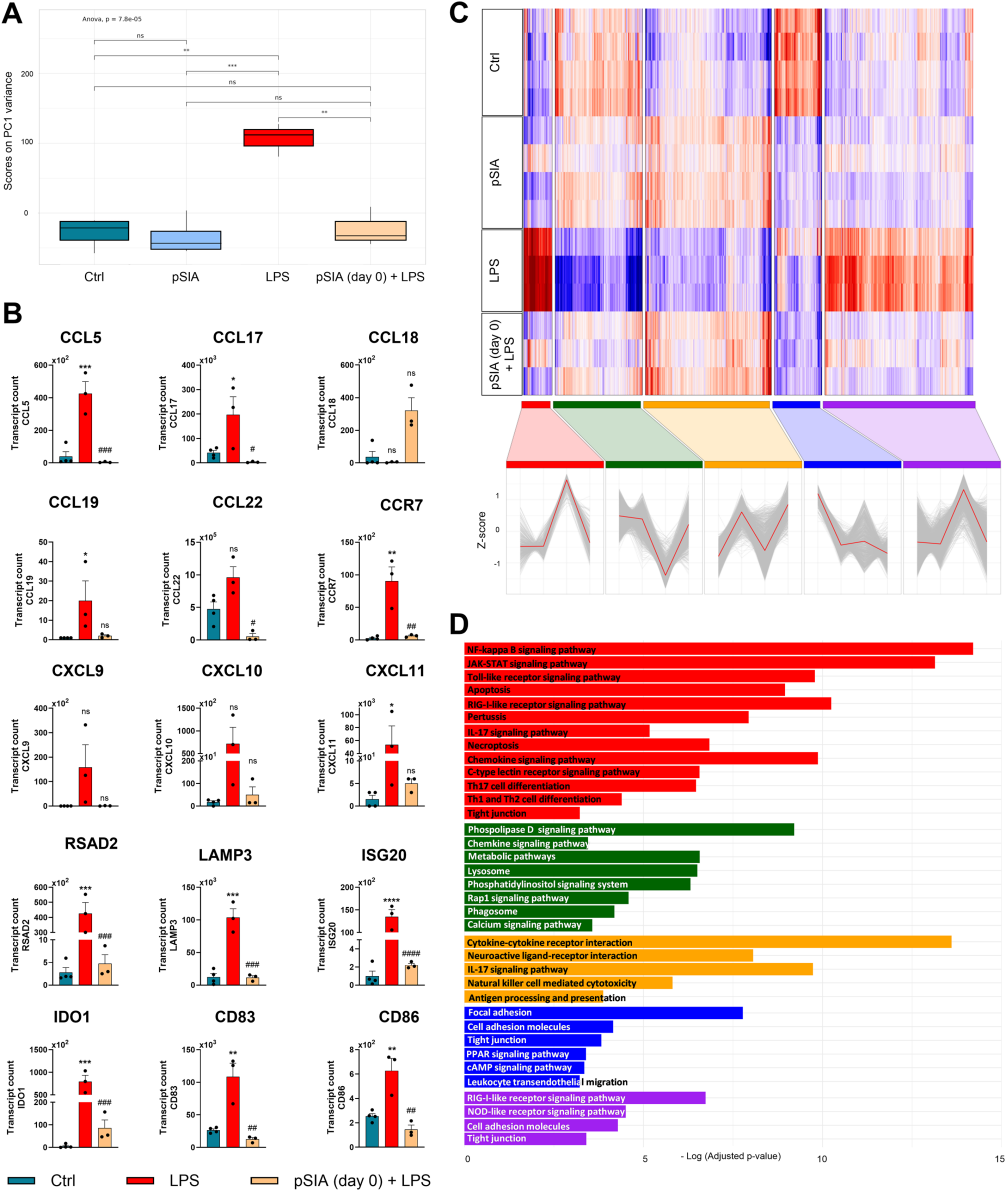
LPS-induced transcriptional changes are inhibited upon exposure to α2.8-polySIA. **(A)** Depiction of first principal component (PC1) variance. **(B)** Representative transcript count analyses of the top 100 genes altered in DCs upon pSIA (day 0) + LPS vs. LPS (without pSIA) conditions. Genes with total counts across samples less than 5 and mean read count < 2 were excluded from the analysis. Only genes with adjusted p-value < 0.05 (Benjamini-Hochberg) were considered as significantly differentially expressed. ns, not significant: p > 0.05, *, p < 0.05, **, p < 0.01, ***, p < 0.001, ****, p < 0.0001 vs. Control group. #, p < 0.05, ##, p < 0.01, ###, p < 0.001, ####, p < 0.0001 vs. LPS group using Kruskal-Wallis test with Dunn’s *post-hoc* test. LPS, lipopolysaccharide; pSIA, α2.8-polySIA. **(C)** Heat map and Z-score transformed expression values (with red and blue indicating up-regulated and down-regulated genes, respectively, compared with the mean value of a gene from all samples) depicting distinct gene clusters associated with treatment groups. **(D)** KEGG analysis showing the pathways associated with the clusters identified in **(C)**.

To further corroborate these results, we profiled transcriptomic changes in the presence or absence of α2.8-polySIA during human monocyte maturation. To this end, CD14^+^ human mononuclear cells were purified from healthy donor-derived PBMCs and underwent standard maturation and activation protocols using GM-CSF, IL-4, and LPS. Bulk RNA sequencing revealed distinct α2.8-polySIA-dependent changes in the transcriptomic landscapes of APCs. The transcriptomic profiles of APC treated with α2.8-polySIA during maturation did not differ from unmatured control groups (**Figure 4A**). By analyzing the top 100 genes altered in the LPS-treated *vs.* LPS+α2.8-polySIA-treated groups, we identified a marked differentiation arrest on a transcriptomic level in α2.8-polySIA-treated APCs. Specifically, treatment with α2.8-polySIA diminished transcripts associated with pro-inflammatory responses and lymphocyte chemotaxis such as *siglec-1*, *cxcl9*, *cxcl10*, ccl22, *ccl17*, and *ccl19*. In contrast, α2.8-polySIA-treatment led to increased expression of genes associated with antigen processing and presentation (*cd209*, *fcgr2b*, *al136295*, and *scl11a1*) or endocytosis (*clec10a*, *asgr2*, *cd14*, *cd163* and *stab1*). The strongest upregulation was observed for both chemokines *cxcl9*, which enhances type 1 DC activation and consequently antigen cross-presentation and T cell activation, and *ccl18*, a chemokine that has been shown to facilitate differentiation of dendritic cells into tolerogenic cells able to prime regulatory T cells (38, 39) (**Figure 4B**). Although the sample size for these analyses was limited, the differences observed between LPS-stimulated cells with and without α2.8-polySIA were consistent across all experiments, corroborating the robustness of the results. Gene clustering and subsequent KEGG analysis for taxonomy-based profiling of pathways revealed distinct α2.8-polySIA-associated changes in APCs (**Figure 4C**; **Supplementary Figure S6**). While *cytokine-cytokine receptor interaction*, *antigen processing, and presentation* pathways were upregulated upon α2.8-polySIA treatment, *leukocyte transendothelial migration*, *focal adhesion*, and *cell adhesion molecule* pathways were downregulated. Additionally, we observed an abrogation in LPS-induced signaling pathways in the polySIA-treated group, which includes NF-Kappa B, JAK-STAT, and Toll-like receptor signaling pathways (**Figures 4C, D**).

**Figure 5:**
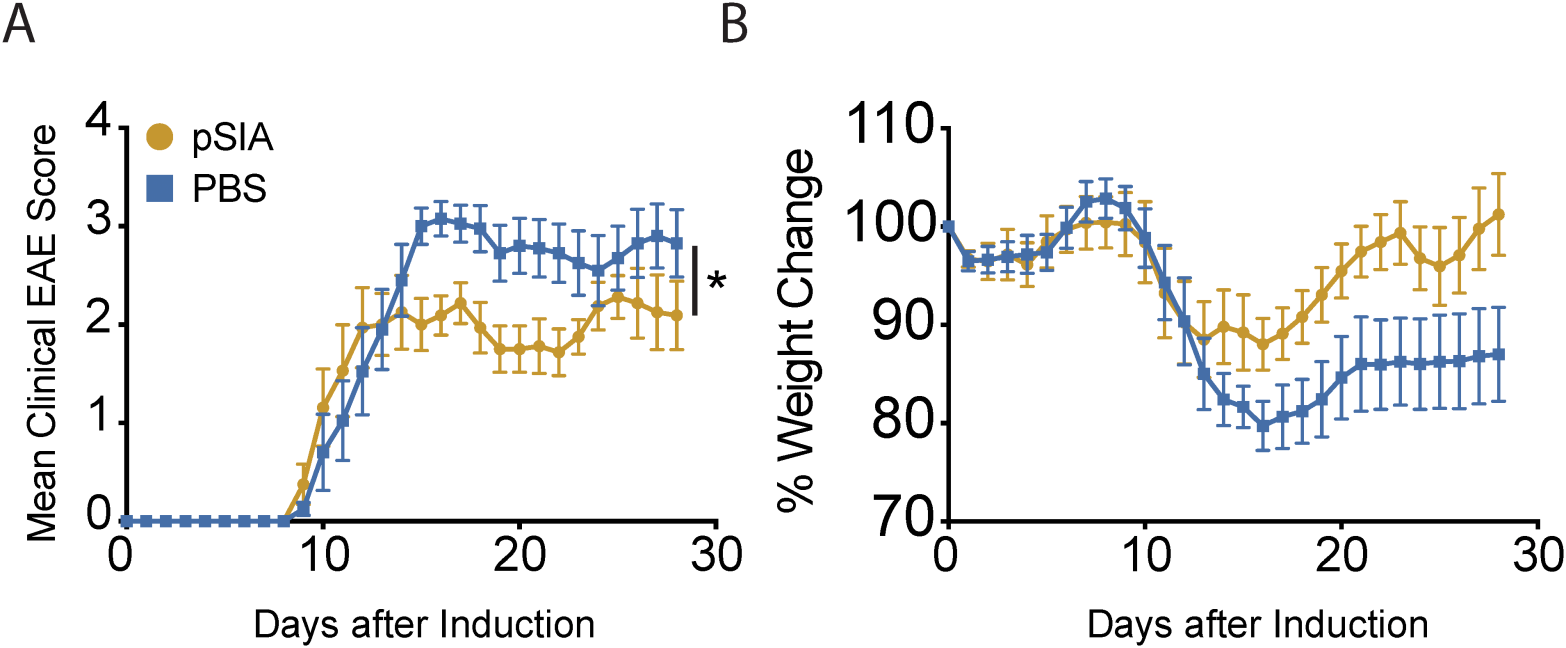
Treatment with α2.8-polySIA during active EAE. **(A)** Mean clinical EAE score over time. **(B)** Percentage of weight loss over time. Each line represents the mean ± SEM for one treatment group (pSIA: n = 10; PBS: n = 8). Data representative of 3 individual experiments are shown. Statistical analysis was performed using two-way ANOVA. Significance is indicated as follows: ns, not significant: P > 0.05, *P < 0.05; **P < 0.005; ***P < 0.001; ****P < 0.0001. pSIA, α2.8-polySIA.

To address whether exposure of CNS-infiltrating myeloid subsets towards polySIA may be harnessed for therapeutic intervention during EAE, we repeatedly treated C57BL/6 mice with i.p. injections of α2.8-polySIA (10 µg/g body weight over 4 consecutive days) after reaching an EAE score of 2 (**Figures 5A, B**). Compared to the vehicle (PBS) control group, mice treated with α2.8-polySIA depicted a milder disease course and showed normalization of body weight (**Figures 5A, B**). Thus, treating C57BL/6 mice early in the disease course with α2.8-polySIA ameliorates further disease progression.

## Discussion

In this study, we investigated the anti-inflammatory mechanisms of action of α2.8-polySIA, a “self” carbohydrate polymer, on monocyte activation and maturation in a preclinical model for CNS autoimmunity and human cell culture systems. We have found that early exposure of murine and human monocytes to α2.8-polySIA, prevented these myeloid cells to fully mature into proinflammatory effector cells adequately equipped with proper antigen-presenting machinery and co-stimulatory receptors. Accordingly, α2.8-polySIA pre-exposed human monocyte-derived antigen-presenting cells depicted impaired capacity to stimulate CD4^+^ T cell proliferation and bulk RNA-sequencing revealed a marked differentiation arrest on a transcriptomic level in α2.8-polySIA-treated APCs. In line with this, *in vivo* treatment with α2.8-polySIA early in the disease course of EAE ameliorated further disease progression.

While the pathogenesis during MS and its animal model EAE is likely to be initiated by auto-aggressive CD4^+^ T cells, focal CNS tissue damage is largely mediated by infiltrating and CNS-resident myeloid cells (22, 40, 41). In particular, the inflammatory milieu of progressive stages during MS is maintained by innate immune responses from blood-borne and autochthonous myeloid cells (40–43). Classical Ly6C^hi^ and non-classical Ly6C^lo^ monocytes are scarce in the healthy brain and have been found to be mostly limited to brain vasculature (44). However, during CNS autoimmunity, bone marrow-derived circulating monocytes are recruited to sites of inflammation where they differentiate into monocyte-derived effector cells, constituting a prerequisite for the onset of clinical symptoms during EAE (45). Phenotypic alterations during local differentiation and the maturation of immature monocytes into neuroinflammatory monocyte-derived cells include the upregulation of MHC class II and costimulatory molecules such as CD40 and CD86 (44, 46, 47). The major component of Gram-negative bacteria cell walls, that is LPS, is widely recognized as a potent activator of monocytes, and its effects include an altered production of key inflammatory mediators, such as TNFα, IL1β, IL6, IL8, IL10, IL12, IL15, and TGFβ (48), most of which have been involved in indirectly mediating tissue damage during CNS autoimmunity. During EAE, auto-aggressive CD4^+^ T cells rely on the local re-encounter of their cognate antigen. The origin and nature of antigen-presenting cells, which locally reactivate myelin-specific T cells, have been controversially discussed. We have found that pre-exposure of immature murine and human monocytes with α2.8-polySIA abrogates LPS-induced differentiation into antigen-presenting cells with upregulated surface expression of MHC class II and costimulatory molecules CD40, CD83, and CD86. Phenotypically, α2.8-polySIA-treated cells resembled cells naïve to LPS-exposure. Furthermore, this α2.8-polySIA-induced phenotypic arrest translated into functional changes, in those human monocytes pre-exposed with α2.8-polySIA were impaired in stimulating CD4^+^ T cell proliferation due to HLA mismatch recognition during mixed lymphocyte reaction indicating not only a phenotypically but also functionally undifferentiated state. In line with this, a recent study showed that murine BMDCs lacking sialic acid expression exhibited an increased ability to induce antigen-specific CD8^+^ T-cell proliferation, not through altered antigen uptake or processing, but likely due to effects on the stability of antigen-presenting molecules—a notion supported by other reports (49, 50).

Under inflammatory conditions several cell subsets (including non-myeloid) have been shown to contribute to antigen-presentation via MHC class II (51, 52). It has been suggested that on-site CNS antigen presentation to myelin-specific T cells can be executed by CNS resident antigen-presenting cells while the progeny of circulating monocytes is less competent in antigen presentation (32, 53–55) and MHC class II expression on CNS-invading CCR2^+^ monocytes is not essential for maintaining inflammatory T cell responses within the CNS (56).

However, neuroinflammatory monocyte-derived effector cells likely propel the inflammatory milieu by mediating tissue damage and the release of pro-inflammatory cytokines may further drive autoimmune T-cell responses beyond cognate interactions (22, 41, 57).

To investigate α2.8-polySIA-dependent alterations in the transcriptomic landscape, we performed bulk RNA sequencing analyses on human monocyte-derived dendritic cells (moDCs). Dimensionality reduction analysis using principal component variance revealed that the maximum variance of differentially expressed genes can be attributed to LPS treatment and that this LPS-dependent transcriptional pattern was fully reversed upon treatment with α2.8-polySIA arresting monocytes in a transcriptionally immature state. By analyzing the top 100 genes altered in the LPS *vs.* α2.8-polySIA+LPS groups, we observed α2.8-polySIA-administration to prevent the increase in genes associated with T cell-attracting inflammatory responses such as *ccl5* (encoding for Rantes), *cxcl9* and *cxcl10*. This chemokine expression pattern is shared by a previously characterized CNS CD11c^+^ dendritic cell population which is located in perivascular clusters and preferentially interacts with neuroinflammatory Th17 cells. Intravital two-photon microscopy analyses revealed a pivotal role of these chemokine-expressing CNS CD11c^+^ cells in the attraction of pathogenic T cells into and their survival within the CNS and depletion of this population led to a marked reduction of encephalitogenic T cell enrichment and ameliorated the EAE disease course (58). Furthermore, CXCL10 is elevated in the cerebrospinal fluid of people with MS (59) and its receptor CXCR3 is highly expressed on the majority of perivascular T cells in MS lesions (60). Interestingly, we found transcripts of the chemokine CCL18 increased upon exposure to α2.8-polySIA. CCL18 has only modest chemoattractant capacity and mainly has been linked to inducing a regulatory and tolerogenic phenotype (61). It is abundantly secreted by immature dendritic cells, preferentially attracts naïve T cells, and is known to induce a CD4^+^CD25^+^FoxP3^+^ regulatory T cell phenotype (62–64). This is in keeping with the notion that exposure to α2.8-polySIA changes the transcriptomic pattern in monocytes beyond arresting the transition into an inflammatory phenotype. When feeding differentially expressed genes into Kyoto Encyclopedia of Genes and Genomes (KEGG) pathway analysis, we found a marked pattern alteration towards downregulation of genes involved in toll-like receptor signaling, NF kappa B signaling, IL-17 signaling, and T_H_17 cell differentiation upon treatment with α2.8-polySIA indicating a less inflammatory and more tolerogenic phenotype. Notably, microglia derived from MS normal-appearing white matter depict impaired CCL18 induction capacity compared to the microglia from healthy donors, suggesting that CCL18 participates in the suppression of local pro-inflammatory immune responses in the CNS (65).

CNS mononuclear phagocytes might represent important targets for irreversible neural tissue damage, particularly during disease progression (40). Current treatment strategies for MS strongly focus on targeting lymphocytes of the adaptive immune system such as T and B cells. However, the effect of MS immunomodulators on myeloid cells is known to contribute to the clinical efficacy of these therapeutic approaches (66, 67). Recently it was demonstrated that myeloid cell-based therapeutic interventions are efficacious in a preclinical model of MS. Specifically, the authors generated monocyte-adhered microparticles for altering myeloid cell phenotype to an anti-inflammatory state through localized interleukin-4 and dexamethasone signals. These modified monocytes lead to decreased levels of systemic pro-inflammatory cytokines, induced modulatory effects on T_H_1 and T_H_17 populations and regulated both infiltrating and tissue-resident myeloid cell compartments with regards to antigen presentation and reactive oxygen species production (68). Beyond EAE, polysialic acid also displays immunomodulatory properties in bacterial infection. In a murine model of pneumococcal pneumonia, loss of polySia on myeloid cells enhanced phagocytosis, altered leukocyte recruitment, and improved pathogen clearance, highlighting its role in regulating innate immune responses (69).

In order to investigate if α2.8-polySIA-induced changes in myeloid cells could be harnessed for therapeutic intervention during CNS autoimmunity, we treated C57BL/6 mice with weigh-adapted i.p. injections of the polymer over 4 consecutive days after reaching an EAE score of 2. Therapeutic treatment after the onset of EAE motor symptoms was considered to be more clinically relevant than administering prophylactic treatment. We acknowledge that our study did not include *in vivo* phenotyping of T cell subsets or monocyte-derived APCs at the peak of EAE following treatment with α2,8-polySIA, which represents a limitation in the mechanistic resolution of our findings. Nonetheless, we observed a significantly attenuated disease course accompanied by normalization of body weight and improved motor performance in treated animals compared to controls. These clinical readouts suggest that α2,8-polySIA exerts a therapeutic effect in EAE. We consider this a valuable basis for future investigations aimed at delineating the underlying immunological mechanisms. While no systematic analysis has determined whether, or to what extent, the polymer crosses the blood–brain barrier, externally administered α2,8-polySia may modulate the systemic immune response—attenuating monocyte and dendritic cell activation, limiting pro-inflammatory cytokine release, and engaging inhibitory Siglec pathways—in turn reducing inflammatory cell infiltration and activation in the CNS.

In conclusion, our data show that the carbohydrate polymer α2.8-linked polySIA potently inhibits toll-like receptor-dependent maturation of monocytes into pro-inflammatory effector cells. The therapeutic efficacy of this compound in the pre-clinical model merits further research into the compound’s potential clinical application.

## Data Availability

The data that support the findings of this study are available from the corresponding author, upon reasonable request.

## Supplementary material

Supplementary Figure 1 Gating strategy used for analysis of BMDCs. CD11c^+^ MHCII^+^ were used to select DCs. Dead cells were excluded with Zombie Acqua Fixable Dye, lymphocytes were gated based on SSC versus FSC and singlets were selected from the FSC-A versus FSC-H dot plot.

Supplementary Figure 2 Gating strategy used for analysis of human moDCs. CD11c^+^ CD209^+^ were used to select DCs. CD14^+^ was used to exclude monocytes. Dead cells were excluded with Zombie Acqua Fixable Dye, lymphocytes were gated based on SSC versus FSC and singlets were selected from the FSC-A versus FSC-H dot plot.

Supplementary Figure 3 Gating strategy used for quantification of CD3^+^ proliferating cells. CD3^+^ CD4^+^ were used to select Th cells. CD8^+^ was used to exclude cytotoxic cells. CFSE dilution was used to quantify CD4^+^ proliferation. Dead cells were excluded with Zombie Acqua Fixable Dye, lymphocytes were gated based on SSC versus FSC and singlets were selected from the FSC-A versus FSC-H dot plot.

Supplementary Figure 4 **(A)** IL-6, **(B)** TNF, **(C)** IL-10 and **(D)** IL-12 levels measured by ELISA in the supernatants of BMDCs culture before (day 5) and after (day 6) LPS stimuli. Five experimental groups were included: untreated DCs (Control; PBS-treated, negative control), DCs treated with α2.8-polySIA alone from day 0 (pSIA), DCs stimulated with LPS alone (LPS; positive control for activation), DCs treated with α2.8-polySIA from day 0 and LPS at day 5 (pSIA (day 0) + LPS) and DCs stimulated with LPS and treated with α2.8-polySIA both at day 5 (LPS + pSIA (day 5)). Results are presented as mean ± SEM. Statistical comparisons were performed using Unpaired t-test (day 5) or the Kruskal–Wallis test with Dunn’s *post-hoc* analysis (day 6). Significance: *, p < 0.05, **, p < 0.01, ***, p < 0.001, ****, p < 0.0001 vs. Control group. ####, p < 0.0001 vs. LPS group. Pooled data from two experiments is depicted. LPS, lipopolysaccharide; pSIA, α2.8-polySIA.

Supplementary Figure 5 **(A)** IL-6, **(B)** TNF, **(C)** IL-10 and **(D)** IL-12 levels measured by ELISA in the supernatants of human moDCs culture before (day 4) and after (day 5) LPS stimuli. Four experimental groups were included: untreated DCs (Control; PBS-treated, negative control), DCs treated with α2.8-polySIA alone from day 0 (pSIA), DCs stimulated with LPS alone (LPS; positive control for activation) and DCs treated with α2.8-polySIA from day 0 and LPS at day 4 (pSIA (day 0) + LPS). Results are presented as mean ± SEM. Statistical comparisons were performed using Unpaired t-test (day 4) or the Kruskal–Wallis test with Dunn’s *post-hoc* analysis (day 5). Significance: *, p < 0.05, **, p < 0.01, ***, p < 0.001, ****, p < 0.0001 vs. Control group. ####, p < 0.0001 vs. LPS group. Pooled data from two experiments is depicted. LPS, lipopolysaccharide; pSIA, α2.8-polySIA.

Supplementary Figure 6 Heat map for the top 100 most highly variable genes upon LPS stimulation, with and without pSIA treatment from day 0.
