# Correction: Polysialic acid restrains inflammatory monocyte maturation

**DOI:** 10.3389/fimmu.2025.1731973

**Published:** 2025-11-20

**Authors:** Ingredy Passos, Benjamin Peschke, Shrey Gandhi, Judith Derdelinckx, Louisa Müller-Miny, Harald Neumann, Jan D. Lünemann, Christian W. Keller

**Affiliations:** 1 Department of Neurology with Institute of Translational Neurology, University Hospital Münster, Münster, Germany; 2 Laboratory of Neuroinflammation, Institute of Experimental Immunology, University of Zurich, Zurich, Switzerland; 3 Department of Immunology, University Hospital Münster, Münster, Germany; 4 Division of Genetic Epidemiology, Institute of Human Genetics, University of Münster, Münster, Germany; 5 Department of Neurology, Faculty of Medicine and Health Sciences, Antwerp University Hospital, Antwerp, Belgium; 6 Laboratory of Experimental Hematology, Vaccine and Infectious Disease Institute (VaxInfectio), Faculty of Medicine and Health Sciences, University of Antwerp, Antwerp, Belgium; 7 Institute of Reconstructive Neurobiology, Medical Faculty and University Hospital of Bonn, University of Bonn, Bonn, Germany; 8 Department of Neurology and Neurophysiology, University Medical Center Freiburg, Freiburg, Germany

**Keywords:** polysialic acid, Siglec receptors, myeloid cells, neuroinflammation, autoimmunity, multiple sclerosis, monocyte maturation

Author Harald Neumann was erroneously assigned affiliation 8: *Department of Neurology and Neurophysiology, University Medical Center Freiburg, Bonn, Germany* by the *Frontiers* journal. This affiliation has now been removed for Harald Neumann. Consequently, affiliation 9: *Department of Neurology and Neurophysiology, University Medical Center Freiburg, Freiburg, Germany* has been renumbered as affiliation 8: *Department of Neurology and Neurophysiology, University Medical Center Freiburg, Freiburg, Germany*.

The original version of this article has been updated.

