# Supplementary figures and images for: Polysialic acid restrains inflammatory monocyte maturation
Source: Front Immunol. 2025 Oct 20;16:1656087. doi: 10.3389/fimmu.2025.1656087 (PMC12580094; doi:10.3389/fimmu.2025.1656087)

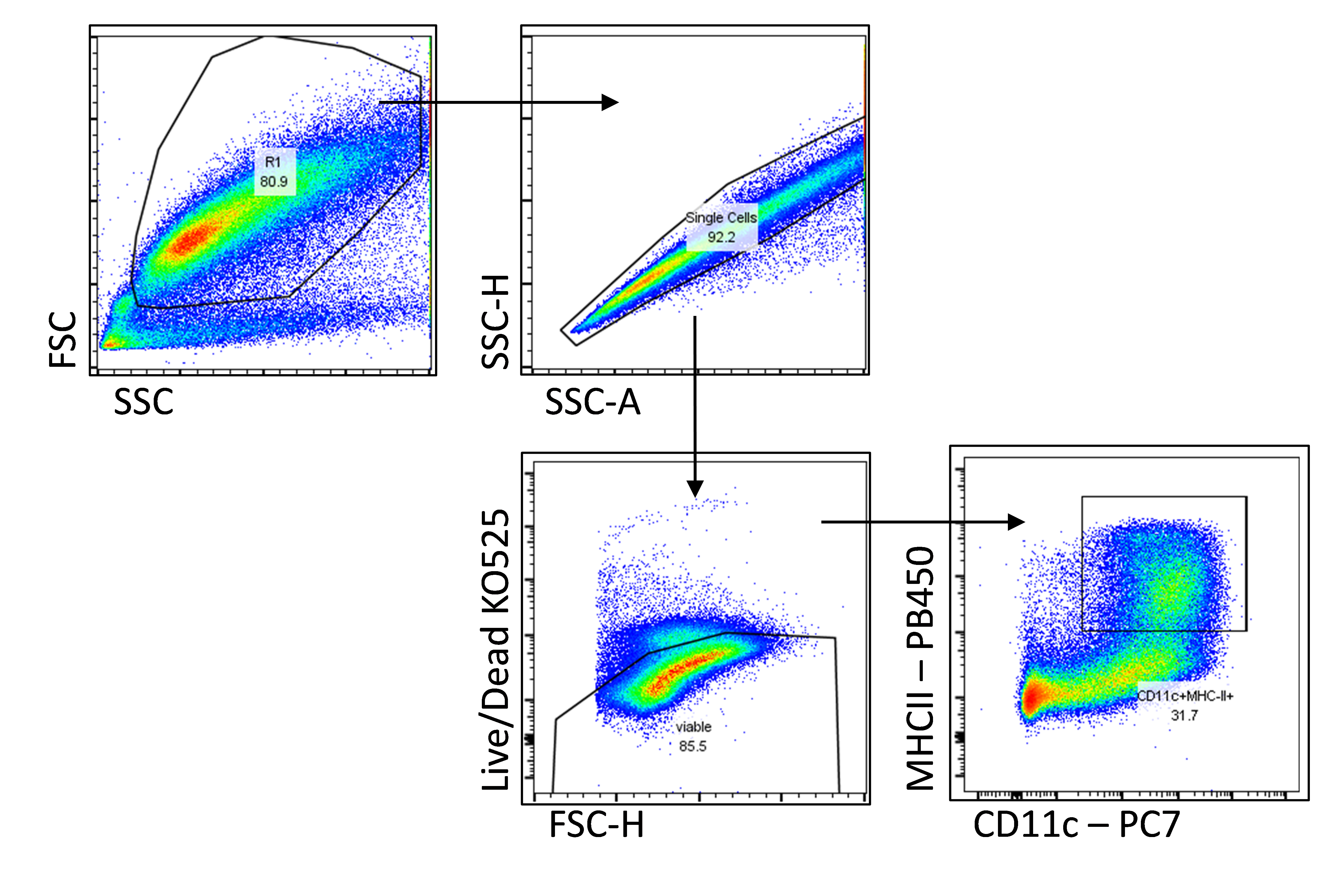

Supplement: Supplementary Figure 1 — Gating strategy used for analysis of BMDCs. CD11c+ MHCII+ were used to select DCs. Dead cells were excluded with Zombie Acqua Fixable Dye, lymphocytes were gated based on SSC versus FSC and singlets were selected from the FSC-A versus FSC-H dot plot. [file Image1.tiff]

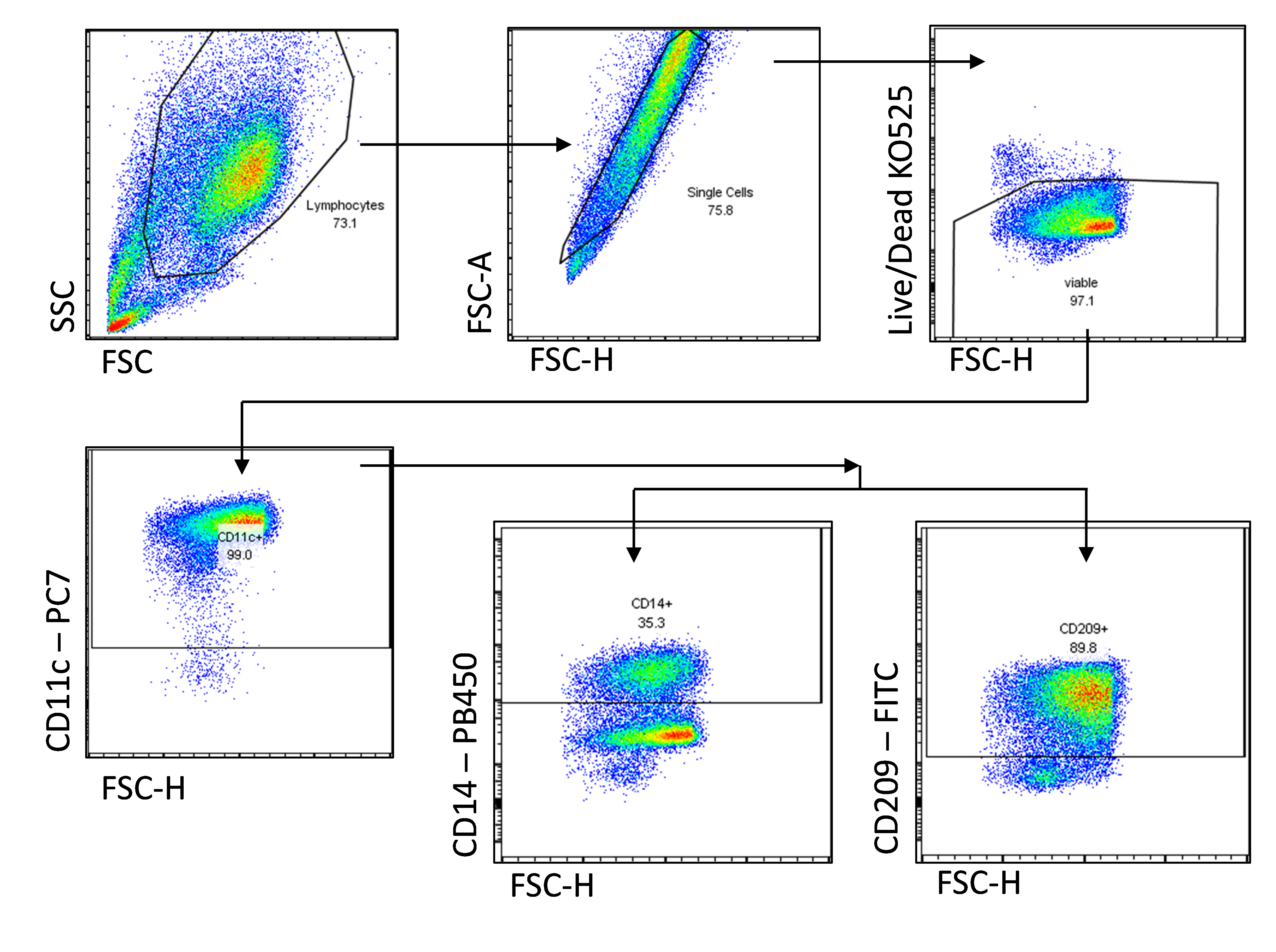

Supplement: Supplementary Figure 2 — Gating strategy used for analysis of human moDCs. CD11c+ CD209+ were used to select DCs. CD14+ was used to exclude monocytes. Dead cells were excluded with Zombie Acqua Fixable Dye, lymphocytes were gated based on SSC versus FSC and singlets were selected from the FSC-A versus FSC-H dot plot. [file Image2.tiff]

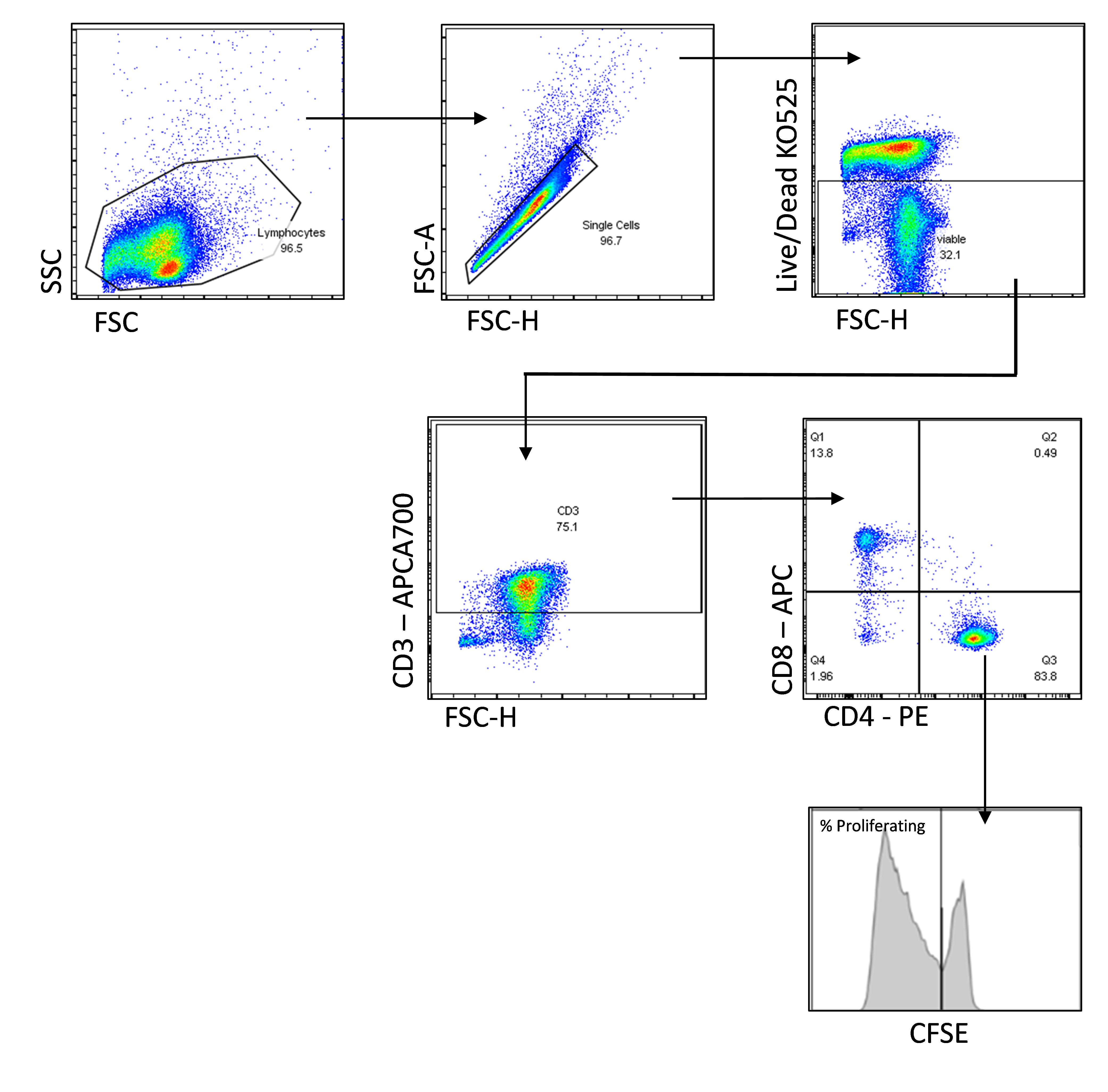

Supplement: Supplementary Figure 3 — Gating strategy used for quantification of CD3+ proliferating cells. CD3+ CD4+ were used to select Th cells. CD8+ was used to exclude cytotoxic cells. CFSE dilution was used to quantify CD4+ proliferation. Dead cells were excluded with Zombie Acqua Fixable Dye, lymphocytes were gated based on SSC versus FSC and singlets were selected from the FSC-A versus FSC-H dot plot. [file Image3.tiff]

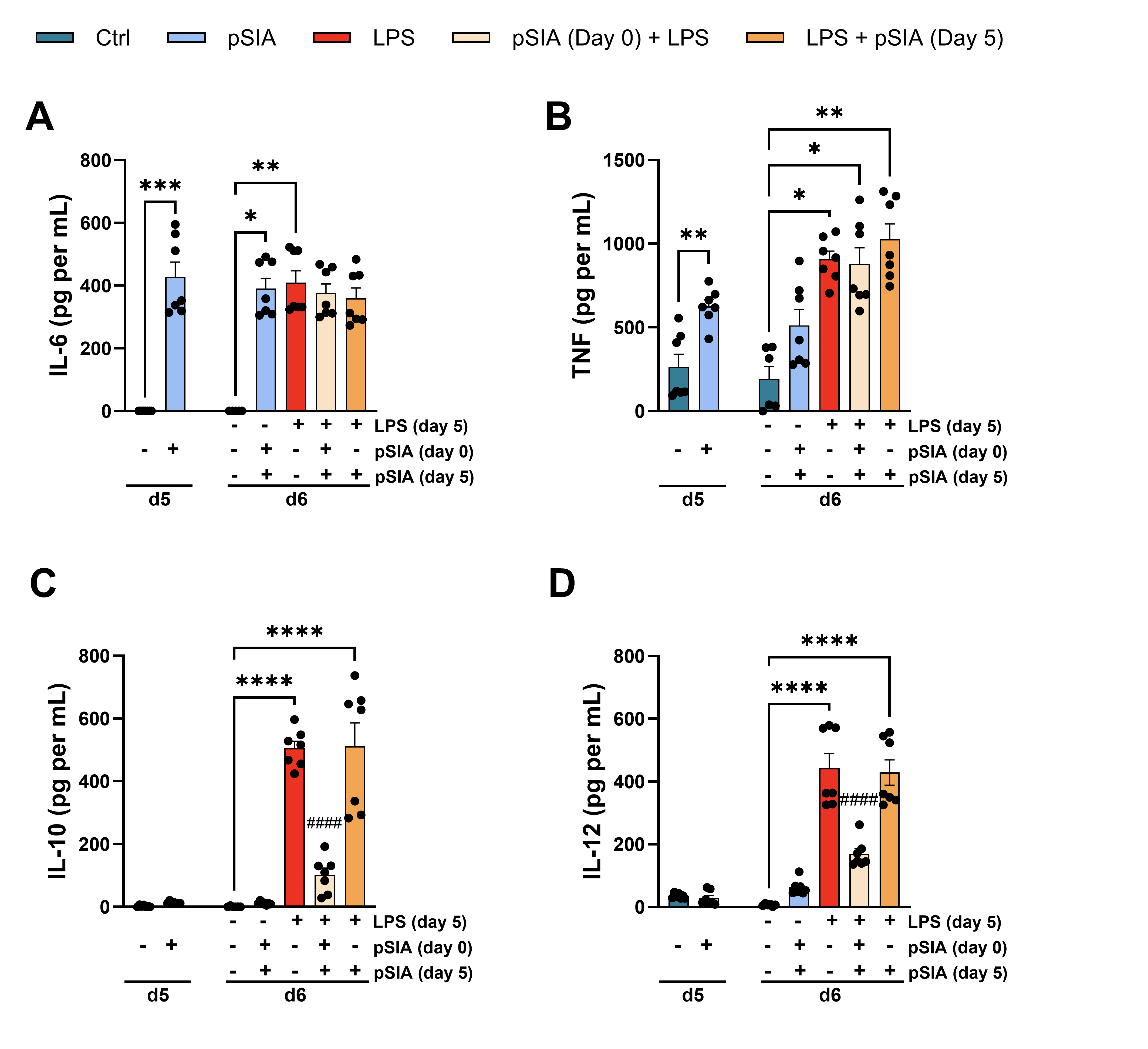

Supplement: Supplementary Figure 4 — (A) IL-6, (B) TNF, (C) IL-10 and (D) IL-12 levels measured by ELISA in the supernatants of BMDCs culture before (day 5) and after (day 6) LPS stimuli. Five experimental groups were included: untreated DCs (Control; PBS-treated, negative control), DCs treated with α2.8-polySIA alone from day 0 (pSIA), DCs stimulated with LPS alone (LPS; positive control for activation), DCs treated with α2.8-polySIA from day 0 and LPS at day 5 (pSIA (day 0) + LPS) and DCs stimulated with LPS and treated with α2.8-polySIA both at day 5 (LPS + pSIA (day 5)). Results are presented as mean ± SEM. Statistical comparisons were performed using Unpaired t-test (day 5) or the Kruskal–Wallis test with Dunn’s post-hoc analysis (day 6). Significance: *, p < 0.05, **, p < 0.01, ***, p < 0.001, ****, p < 0.0001 vs. Control group. ####, p < 0.0001 vs. LPS group. Pooled data from two experiments is depicted. LPS, lipopolysaccharide; pSIA, α2.8-polySIA. [file Image4.tiff]

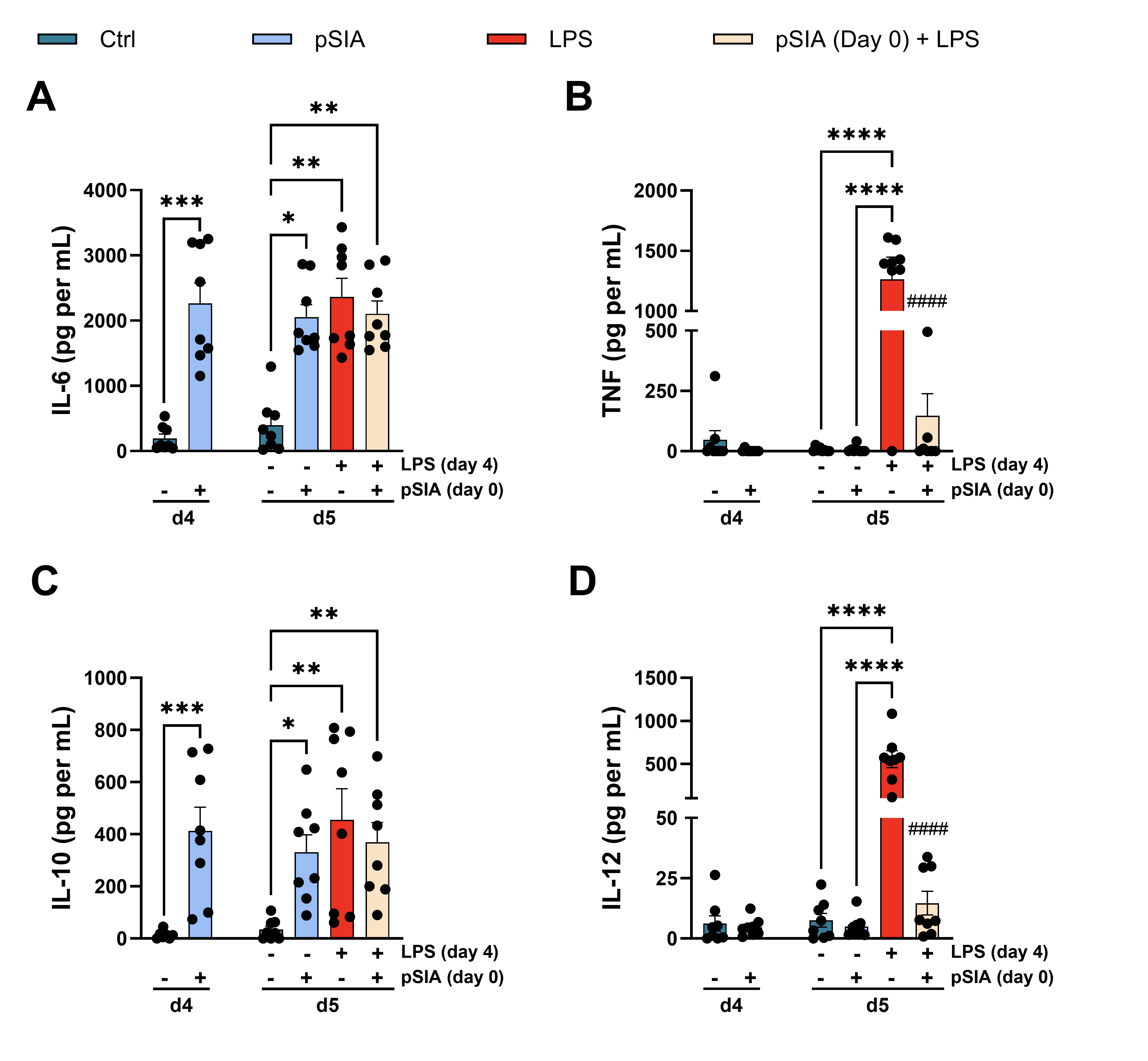

Supplement: Supplementary Figure 5 — (A) IL-6, (B) TNF, (C) IL-10 and (D) IL-12 levels measured by ELISA in the supernatants of human moDCs culture before (day 4) and after (day 5) LPS stimuli. Four experimental groups were included: untreated DCs (Control; PBS-treated, negative control), DCs treated with α2.8-polySIA alone from day 0 (pSIA), DCs stimulated with LPS alone (LPS; positive control for activation) and DCs treated with α2.8-polySIA from day 0 and LPS at day 4 (pSIA (day 0) + LPS). Results are presented as mean ± SEM. Statistical comparisons were performed using Unpaired t-test (day 4) or the Kruskal–Wallis test with Dunn’s post-hoc analysis (day 5). Significance: *, p < 0.05, **, p < 0.01, ***, p < 0.001, ****, p < 0.0001 vs. Control group. ####, p < 0.0001 vs. LPS group. Pooled data from two experiments is depicted. LPS, lipopolysaccharide; pSIA, α2.8-polySIA. [file Image5.tiff]

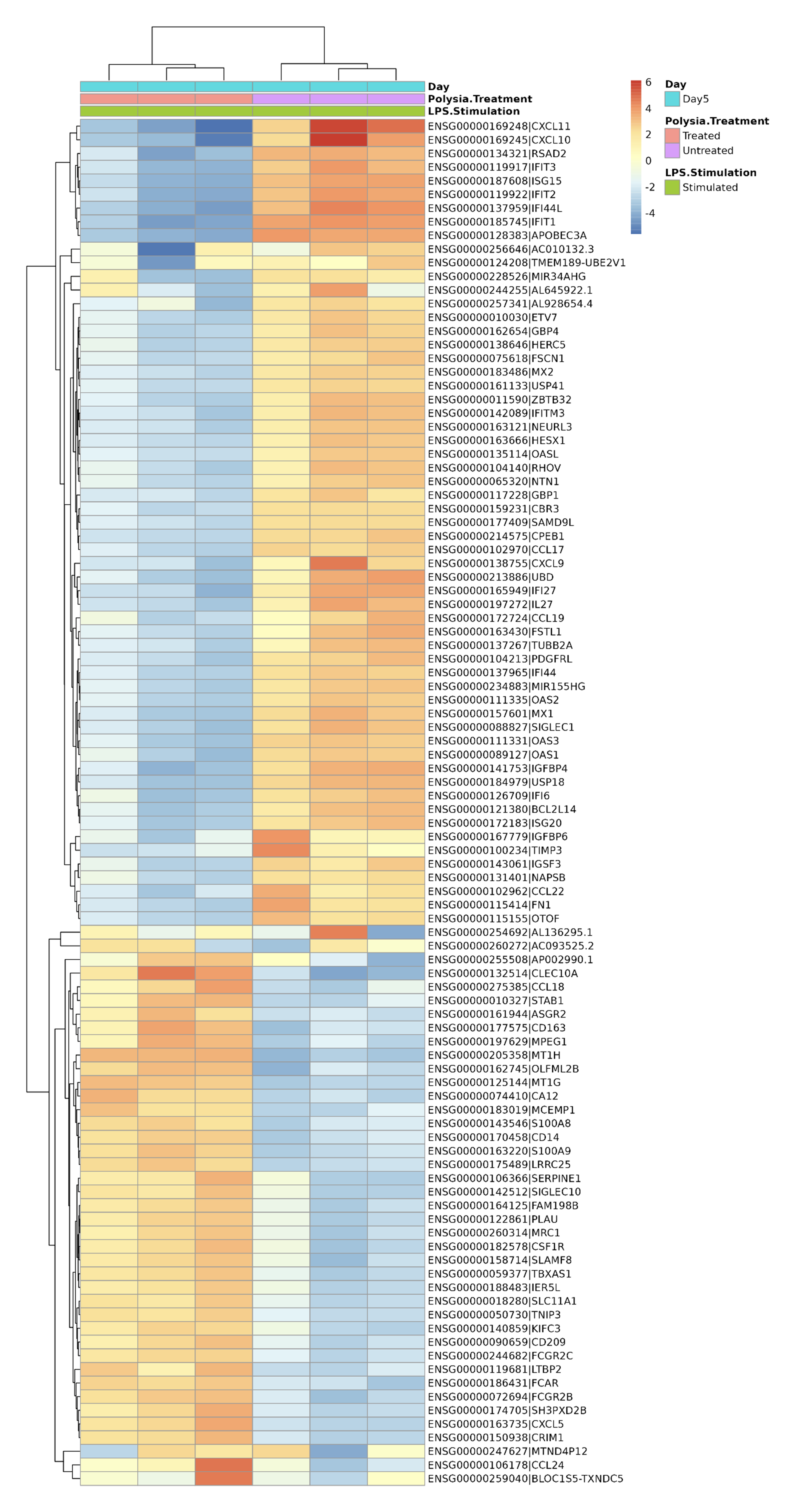

Supplement: Supplementary Figure 6 — Heat map for the top 100 most highly variable genes upon LPS stimulation, with and without pSIA treatment from day 0. [file Image6.tiff]
